# Preserved Imitation of Known Gestures in Children with High-Functioning Autism

**DOI:** 10.1155/2013/751516

**Published:** 2013-08-25

**Authors:** Joana C. Carmo, Raffaella I. Rumiati, Roma Siugzdaite, Paolo Brambilla

**Affiliations:** ^1^Area of Neuroscience, SISSA, Via Bonomea 265, 34136 Trieste, Italy; ^2^Faculty of Psychology, University of Lisbon, Alameda da Cidade Universitária, 1649-013 Lisboa, Portugal; ^3^Medical Image and Signal Processing Group, Department of Electronics and Information Systems, Ghent University—iMinds, De Pintelaan 185, 9000 Ghent, Belgium; ^4^Department of Experimental and Clinical Medical Science (DISM), University of Udine, P.le Kolbe 3, 33100 Udine, Italy; ^5^IRCCS “E. Medea” Scientific Institute, UDGEE, P.le S. Maria della Misericordia, 15 Udine, 33100 Udine, Italy

## Abstract

It has been suggested that children with autism are particularly deficient at imitating novel gestures or gestures without goals. In the present study, we asked high-functioning autistic children and age-matched typically developing children to imitate several types of gestures that could be either already known or novel to them. Known gestures either conveyed a communicative meaning (i.e., intransitive) or involved the use of objects (i.e., transitive). We observed a significant interaction between gesture type and group of participants, with children with autism performing known gestures better than novel gestures. However, imitation of intransitive and transitive gestures did not differ across groups. These findings are discussed in light of a dual-route model for action imitation.

## 1. Introduction

The relationship between autism and imitation deficits was envisaged long time ago by Ritvo and Province [1], short after autism was originally described in the work of Kanner [2]. The imitative ability has been acknowledged to play an essential role in normal development, as it can be used by infants to acquire and master new behaviors [3, 4]. In recent years, the relationship between autism and voluntary imitation has been investigated more systematically. In their review, for instance, Williams et al. [5] concluded that children with an autism spectrum disorder (ASD) are consistently impaired in performing imitative tasks relative to children with other developmental delays (matched for chronological age, verbal IQ-mental age and expressive language (see [6, 7]), or to normal controls matched for mental age [7, 8]. ASD children performed more poorly on imitation tasks than matched-to-language controls or younger children matched for receptive language and mental age [8], thus ruling out the interpretation that the imitation impairment is due to a linguistic deficit. 

Neither can the imitative deficit of ASD individuals be attributed to a defective gesture recognition [6, 8], since they recognized gestures without any trouble. Moreover, ASD individuals' fine grained motor skills, even when reduced, did not correlate with their observed imitation and praxis deficits [8]. 

Moreover, irrespective of whether they are low or high functioning, children with autism seem to have difficulties in imitating gestures that disable children and typically developing children do not show [9]. This finding supports the view that imitation deficits are specific to ASD (see also [5]).

Whether the reduced ability to imitate of ASD children is a true deficit or a delay in development is still under debate. This deficit seems to be already present in children as young as 20 months and is, in general, more apparent in children than in adults [5]. Consistently, an improvement in performance was observed when young ASD children, with a mean age of 31 months, were retested about one year later [7].

Williams et al. [5] proposed that observing a meaningful (MF) object or gesture triggers the release of a previously rehearsed program or that observing a desired outcome might lead the observers to reach this goal by applying their own problem solving ability. This process is known as emulation. Hence, imitation itself would be especially required for copying meaningless (ML) gestures that do not have an obvious goal or associated knowledge [5]. This would explain why in some studies ASD children were reported to experience more difficulty when imitating ML compared with MF gestures involving objects [6, 7].

It has been shown that ASD children imitate goal-directed actions as well as healthy children with the same verbal mental age do [10]. Hamilton et al. [10] argued that the ability to imitate (and understand) the goal of hand actions is intact in ASD children who, however, might fail to perform the imitation tasks in which they cannot rely on a hand-goal strategy as in the case of ML actions. They also suggested that it is unlikely that a unique neurocognitive mechanism underlies imitative behavior in either the typical or autistic brain. Consistently with this view, Hamilton [11] put forward a dual-route model for emulation and planning versus mimicry (EP-M model) and proposed that the latter is impaired in children with autism. According to this model, the mirror neuron system is fractioned into an indirect, parietal route for goal emulation and planning and a direct occipitofrontal route for mimicry. In the case of the E-P route, the presence of an object or goal allows an individual to extract the teleological understanding of the action and to reconstruct the same action by his/her own means. The M-route is based on the analysis of low level kinematics of an action and allows the formation of direct associations from visual input to motor output [11].

However, whether children with ASD are able to imitate communicative gestures (such as waving a hand for goodbye) is still debated. Communicative gestures, even though they have been included in the assessment of more than one study, have either been analysed together with other gesture types (e.g., [7]) or, when analysed as a separate class, they were found to be imitated normally [6]. This latter result is difficult to interpret because, in addition to the instruction to imitate, participants were also provided with verbal encouragement (e.g., “Show someone that you are a champion”). Moreover, different studies used different tasks and included participants of different ages (for children see [8]; for adolescents see [6]; for adults see [7]). 

If, according to the EP-M model, children with autism have a selective damage to the mimicry route [10, 11], one would predict that they should be equally impaired at imitating novel as well as symbolic, communicative gestures. In fact, as these two types of gestures do not have an obvious outcome, children cannot rely on an emulation and/or problem solving. A different set of predictions can be drawn based on a dual-route model of action imitation [12] (see also [13, 14]) that is also consistent with the conclusions reached by Williams et al. [5]. The key feature of Tessari and Rumiati's model is the presence of two different mechanisms subserving imitation: a direct route necessary for reproducing novel, ML actions, as it translates the visual input into a motor output and an indirect route that can be used only for imitating over-learned, MF actions, as it accesses the stored representations of the motor act. Since both symbolic communicative (i.e., intransitive) and transitive gestures (i.e., actions that involve the use of objects) are already known by the subjects, imitation of either type of gestures should be comparable in terms of accuracy. After brain damage, each mechanism can be selectively impaired, giving rise to different imitation deficits affecting either known or novel actions [15–18]. In particular, when the direct route is damaged, patients cannot imitate novel actions, while when the indirect route is damaged, they cannot use it to imitate known actions. These findings were observed when known, and novel actions were presented in separate lists. 

In the present study, we aimed to clarify how high-functioning autistic (HFA) children imitated different types of actions (i.e., known MF symbolic communicative, pantomime of object use, and novel ML actions) and to test which of the available models best accounts for their impaired-preserved imitative pattern. Importantly, we consider that investigating imitation performance in ASD is highly valuable as it allows testing current theoretical models of imitation.

## 2. Methods

### 2.1. Participants

Thirteen high-functioning children with autism (M = 7.31 years old, SD = 1.79; all males) and 14 typically developing children (M = 7.00 years old, SD = 1.71; all males) participated in the study. Age did not differ between the two groups (*t*(26) = 0.46, *P* > 0.1). All children were right-handed (average on the percentage of right hand use HFA: M = 90.91%, SD = 15.02, controls: M = 89.89%, SD = 16.62). All autistic participants were clinically diagnosed as having a pervasive development disorder—not otherwise specified—according to DSM-IV. None of the patients had comorbid attention deficit and hyperactivity disorder (ADHD), seizure disturbance, or any other associated disorder known to cause autism. Intelligence scales and autism specific scales were administered by each child responsible therapist. High-functioning autistic participants were selected if they had a full scale IQ > 70 and they scored above threshold for autistic spectrum disorder on the children autistic rating scale (CARS) or ADOS (see Table 1). Children with autism were recruited through the neuropsychological unit at “La Nostra Famiglia” (Pasian di Prato, Udine, Italy). Typically, developing children were recruited from local schools and were administered an intelligence scale (WISC, verbal subscale) by the experimenter. Ethical permission for the study was granted by SISSA ethical committee, and informed consent was given by one parent of each child.

### 2.2. Imitation Task

All children attended the experimental session for approximately 1 hour. Stimuli consisted of five sets of 12 simple, nonsequential gestures each. Of these, three sets included MF gestures: 12 transitive gestures *without* objects like, for instance, pretending to pour from an imagined bottle (all taken from [12]), 12 intransitive symbolic gestures like, for instance, waving goodbye, “victory,” and “come here” (all taken from [19]), and 12 transitive gestures performed *with* an object like, for instance, pouring from a real bottle. The remaining two sets included 24 meaningless (ML) gestures, obtained by modifying the relationship between hand, arm, and trunk of the MF transitive and intransitive gestures. ML and MF actions were as much as possible matched for complexity. Each action was displayed up to two times for 3 seconds on a computer screen using Presentation software (Neurobs). All actions were modelled by a female adult using her right hand arm; subjects were only given the instruction “do what she does” without mentioning the hand they should use. When performing the gestures, the model kept the gaze fixed straight ahead, thus avoiding confound effects regarding the possibility of reading intentions from gaze [20].

Participants' performance was video-recorded and scored offline by a rater, blind to the predictions of the study and to group membership, who was instructed to code each action using a 3-point scale system: 0 for totally incorrect, 1 partially correct, and 2 for correct imitation. For the partially incorrect imitative performance, the rater was asked to code the errors with one of 15 *a priori* defined error-types (see the appendix) and report the hand used in each trial. Participants could begin the experimental session either with MF (3 blocks) or ML gestures (2 blocks), and this order was counterbalanced across participants. An additional rater scored the performance of approximately 30% of the participants, and a good interraters agreement was obtained (Cohen's Kappa = 0.65, S.E. = 0.052).

Two types of assessment were carried out in order to ascertain what participants knew about the intransitive and transitive gestures employed in the study. For the symbolic intransitive actions, participants were asked to say whether they knew the meaning of each action (*n* = 12); for the transitive gestures, participants were presented with the corresponding object and asked to demonstrate how it is normally used. This latter task allowed us also to evaluate participants' hand dominance, given that no instructions were provided to the children as to which hand they should use with the object placed on the table in front of them. The order of the imitative tasks and of the knowledge assessment tasks was counterbalanced across subjects.

## 3. Results

### 3.1. Imitation

For each participant, MF (transitive or intransitive) gestures that were not recognized were not included in the analysis. HFA children were able to recognize on average 74.36% (SD = 14.22) and control children 85.61% (SD = 5.16) of the intransitive gestures. HFA children were able to demonstrate the use of objects (transitive actions) on average 98.71% (SD = 3.13) and control children 100%.

**Figure 1:**
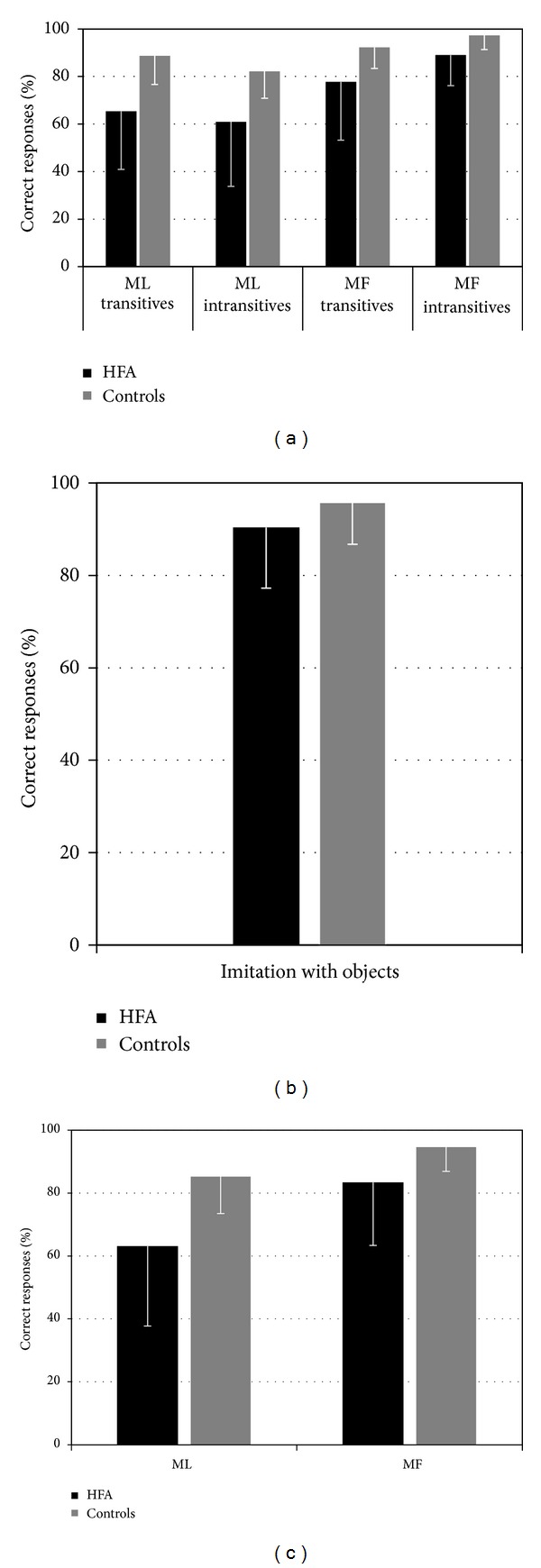
Accuracy on the imitative tasks of the experimental HFA group (in black) and control group (in grey). The bars represent standard deviations from the mean. Upper plot depicts correct imitation of meaningless (ML) and meaningful (MF) according to whether they are transitive or intransitive.

A repeated-measures ANOVA on percentage of imitative correct responses with meaning (ML, MF) and context (transitive, intransitive) as within-subjects independent variables and with group (HFA, controls) as a between-subjects variable (see Figure 1) was performed. Overall, the HFA group imitated more poorly (M = 73.26, SE = 3.96) than the control group (M = 90.10, SE = 3.82); meaningful actions (M = 89.10, SE = 2.22) were performed better than meaningless (M = 74.28, SE = 3.54), and the accuracy with intransitive actions (M = 82.33, SE = 2.72) and transitive actions was comparable (M = 81.01, SE = 3.32). Main effects of group and meaning were found to be significant (*F*(1, 26) = 9.355, *P* = 0.005; *F*(1, 26) = 45.84, *P* < 0.001, resp.) but not the main effect of context (*F*(1, 26) = 0.26, *P* > 0.05). As predicted, the two-way interactions group × meaning (*F*(1, 26) = 6.21, *P* < 0.05) were significant but not the two-way interaction group × context (*F*(1, 26) = 0.26, *P* > 0.05) nor the 3-way interaction (group × context × meaning: *F*(1, 26) = 0.35, *P* > 0.05). Context × meaning interactions were also found significant (*F*(1, 26) = 14.59, *P* = 0.001 resp.).

In order to better understand the interactions, we performed subsequent post hoc analysis. As to the meaning × group interaction, no differences were found between HFA and control children in imitation of MF actions (Tukey, *P* > 0.1), whereas differences were found for imitation of ML actions (Tukey, *P* < 0.05). The difference between imitation of MF and ML actions is driven mostly by HFA children who imitated ML actions more poorly (Tukey, *P* < 0.001). Control children imitated MF actions somewhat better than ML actions (Tukey, *P* = 0.024, see Figure 1). Regarding the Context × Meaning interaction, no significant differences were found in imitating transitive and intransitive ML actions (Tukey, *P* > 0.1), while intransitive MF actions were found to be better imitated than transitive MF actions (Tukey, *P* = 0.019). However, the context × meaning interaction seems to be mostly driven by differences in imitating intransitive actions: imitation of intransitive MF actions and imitation of intransitive ML actions were highly significant (Tukey, *P* < 0.001) (with Bonferroni correction for *P* values).

The two groups did not significantly differ as far as imitation of actions involving real objects was concerned (*F*(1, 26) = 1.59, *P* > 0.05). Importantly, we found no significant correlation between individual IQ quotients and the imitative performance on neither ML actions (*r* = 0.34, *P* > 0.05) nor MF actions (*r* = 0.32, *P* > 0.05).

In order to assess whether children IQ level influenced their individual imitative performance, we computed an analysis of covariance (ANCOVA) with group (HFA, controls) and IQ scores as predictors. The covariate variable (IQ) was standardized prior to performing the analysis, by centering the mean (see [21]). Consistent with the correlational analysis, we found that overall IQ level did not influence participants imitative performance (*F*(1, 24) = 2.362, *P* > 0.1), not even when the meaning of actions was taken into account (IQ × meaning interaction: *F*(1, 24) = 0.232, *P* > 0.6). The other factors did not change.

### 3.2. Does Age Matter?

**Figure 2:**
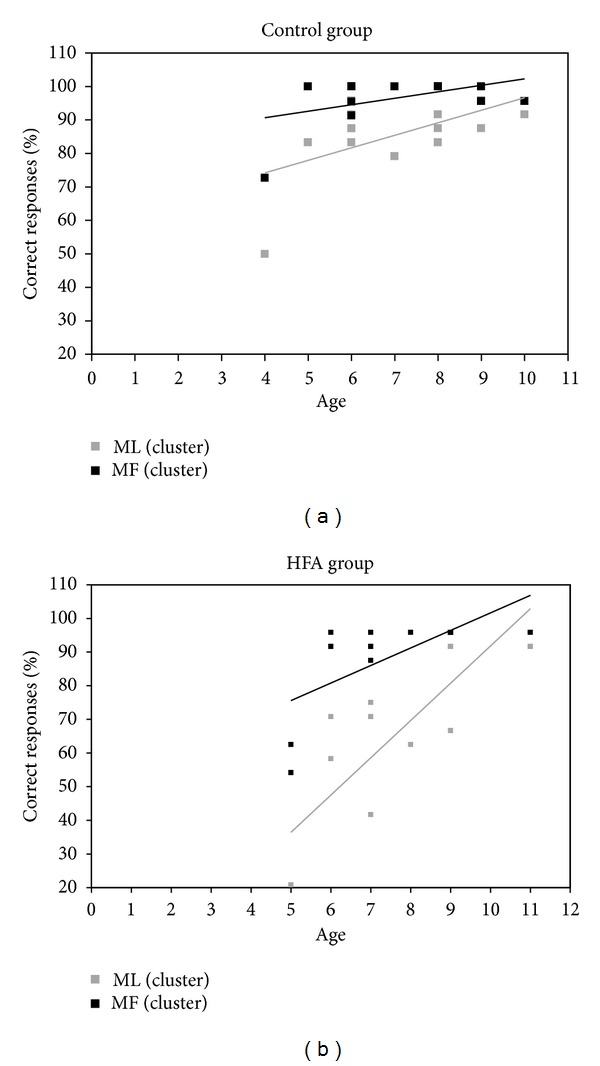
Overall imitation of ML and MF actions correlated with age of subjects in (a) the control group and (b) the HFA group.

We correlated the performance on imitation of MF and ML actions, regardless of whether they were transitive or intransitive, with the age of the control participants and of the HFA children, respectively (see Figure 2). We found that the imitative performance of the control group on ML actions (but not that of meaningful actions, *r* = 0.51, *P* > 0.05) significantly correlated with increasing subjects' age (*r* = 0.56, *P* < 0.05), while the imitative performance of the HFA group on either type of gestures significantly correlated with their increasing age (MF: *r* = 0.72, *P* < 0.05; ML: *r* = 0.79, *P* < 0.001). Due to a clear outlier in the control group (a child with 4 years old), we have recalculated the correlations regarding the control group excluding this participant. In contrast with the HFA group, the results show now that neither the performance of ML actions nor of MF actions correlated with the age of participants (MF: *r* = −0.06, *P* > 0.55; ML: *r* = 0.29, *P* > 0.05).

### 3.3. Specular and Anatomical Imitation

Participants could imitate a gesture by selecting the same limb used by the model (i.e., anatomical imitation) or by using the one on the same side of the model's body as if they were looking in a mirror (i.e., specular imitation). It has often been claimed that typically developing children at a young age tend to prefer specular over anatomical imitation and that the preference becomes apparent when they are about 12 years old [22]. Which kind of imitation ASD children prefer is still not clear. In one study, unlike controls, adults with autism did not benefit from viewing other person's mirror-image movements [23], while in another one, both control and ASD children showed a preference for mirror imitation of hand actions [10]. In these studies, since the model performed the action using the left hand in half of the trials while the imitator tended to use predominantly the dominant right hand, it is difficult to establish whether participants preferred the specular to the anatomical imitation. 

**Figure 3:**
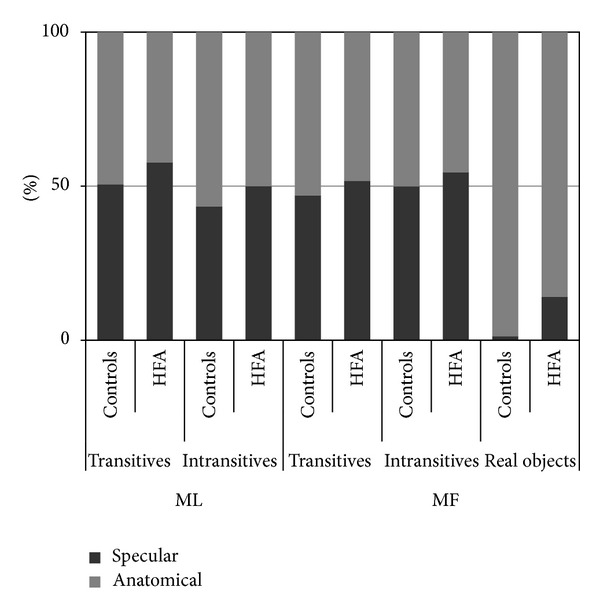
Percentage of specular and anatomical imitations for each type of imitative tasks.

Figure 3 plots the percentage of trials in which participants used the left hand (specular imitation) to imitate the different types of gestures. Although the autistic group made more use than the control group of specular imitation in all imitative tasks, on the Mann-Whitney *U* test for independent samples, no significant differences were found between the two groups on imitation of transitive (*U* = −0.46, *P* > 0.1) and intransitive ML gestures (*U* = −0.20, *P* > 0.1), transitive with (*U* = −1.6, *P* > 0.1) and without (*U* = −0.23, *P* > 0.1) the actual object at hand, and intransitive MF gestures (*U* = −0.41, *P* > 0.1).

## 4. Discussion

In the current study we aimed to understand whether children with autism have a generalized deficit in imitating all gesture types or they only fail with object nonrelated gestures. We therefore tested a sample of high-functioning children with a clinical diagnosis of autism and an age-matched sample of typically developing children for their ability to imitate either novel or known gestures. Gestures could, or could not, involve the presence of an object (transitive or intransitive). In agreement with Williams et al. [5] view, we found that, relative to control children, high-functioning autistic children were more impaired in imitating novel than known actions. In addition, we found that the imitative performance of either group of participants was not dependent on whether the gesture implied the use of an object or not (transitive versus symbolic communicative), as both MF transitive and symbolic communicative gestures were imitated equally well by both groups. 

In particular, the finding that imitation of object-related actions as well as symbolic communicative gestures is preserved in ASD children is inconsistent with the hypothesis put forward by Hamilton [11]. According to this author's proposal, children with autism are able to imitate only gestures that imply the use of an object (or include a clear goal) as its presence allows the emulation of the action instead of mimicry. An individual emulates an observed action by first extracting the goal of the action and then planning and reconstructing it by its own means [11]. The fact that HFA and control children imitate equally well MF actions that do not have an object associated (i.e., symbolic communicative gestures) challenges the view that autistic children are only able to emulate. 

The finding that, in HFA children, imitation of both transitive and intransitive MF actions is intact while imitation of ML actions is impaired strongly suggests that they suffer from a selective damage to one of the two putative mechanisms for action imitation hypothesised by Tessari and Rumiati [12]: the direct route. The access to the representational system for actions stored in memory appears to be intact in these children. 

Our results could possibly have useful implications for an early diagnose of the disorder, in that, we suggest that only the use of ML actions is appropriate for assessing imitation skills. Due to several factors, the diagnosis of autism is still often made quite belatedly (around 6 years of age [24]) because it is based on the assessment of language skills that do not develop before the first year of life. Our study suggests that testing imitation could be used for identifying the eventual presence of the disorder without having to wait for the emergence of linguistic abilities, particularly in high-functioning children. The fact that HFA children imitated MF actions that convey a communicative meaning just like the control group did is apparently in contrast with the view that autism is primarily associated with social deficits (e.g., [25]). According to this position, and in contrast to our data, it should be expected that the stimuli that pose difficulties for autistic children are those that carry emotional or social content. Although any type of imitation procedure involves the interaction between the child and another person, not all imitation procedures seem to be impaired in ASD children. What is striking about our results is that, while the gestures that explicitly carried social or communicative content were correctly imitated by the ASD children, the gestures that did not have any special social content were not correctly imitated. As imitation processes have been acknowledged to play an important role in empathy, for they promote shared mutuality in social interactions and force social bonding [26], the social deficits in autism, such as poor interaction with others or retraction from social interaction, might be secondary to and develop as in consequence of the imitation deficit described in ASD. Regarding imitation of both ML and MF gestures, the accuracy of autistic children tended to improve with participant's increasing age. The recovery of symptoms in ASD has long been debated and mechanisms that might promote it have been systematically scrutinized (see [27] for a review). Although our results cannot be taken as definitive evidence, they are in agreement with the hypothesis that the imitative ASD children's difficulties might not represent a true deviance but rather a delay in development, as they ameliorate as children get older. Therefore, the imitative deficit could be expected to back down as the child gets older.

In the present study, we evaluated the ability to imitate of high-functioning autistic children using a voluntary imitation procedure rather than an automatic imitation paradigm (see e.g., [28]), whereby participants show a facilitation effect (shorter reaction times, RT) when executing a prefixed movement at the same time as they observe the same movement (compatible trials); in contrast, observing a movement incompatible with the preinstructed one leads to an interference (longer RT). This paradigm has been suggested to tap the mirror neuron system in humans [29]; as performance on it has been found to be preserved in ASD [30], it is not clear whether ASD individual performs poorly on imitation tasks due to a malfunctioning mirror neuron system [31]. Although we do not directly tackle automatic imitation in the present study, we predict that an abnormal mirror neuron system would affect primarily actions that are already present in one's own repertoire (known actions) [32].

We failed to find any difference between specular and anatomical imitation. In approximately half of the trials, participants did prefer to imitate with their nondominant hand, mirroring the observed movement. Overall, our results suggest that, for both autistic and typically developing children, imitating an observed movement in a specular manner might be beneficial.

In conclusion, our main findings on the imitative performance of HFA children inform us of a dissociation between imitation of MF actions and imitation of ML novel actions. This fact is rather suggestive of a preserved indirect memory-based mechanism to imitation together with a potentially malfunctioning direct route to imitation.

## Figures and Tables

**Table 1:** Demographical information about the two groups and their performance on diagnostic standardised tests. Mean (standard deviation) and range are provided for each group.

	*N *	Age	Verbal IQ (WISC, Griffiths)	Performance IQ (WISC, Griffiths)	Full scale (WISC, Griffiths, or Leiter)	CARS	ADOS
Autistic group	13	7.31 (1.79) 5–11	86.91 (21.61) 54–116 (*n* = 11)	89.92 (19.53) 66–126 (*n* = 12)	88.58 (17.72) 70–118 (*n* = 13)	42.35 (4.18) 35.5–48 (*n* = 10)	12.33 (6.66) 8–20 (*n* = 3)

Control group	14	7 (1.71) 4–10	127.38 (25.10) 92–156	—	—	—	—

## Appendix

### Imitative Performance Error Type

1. Spatial error of the hand: the overall movement of the limb is correct, but the hand posture is wrong.
2. Spatial error of the arm: the arm posture is wrong.
3. Spatial error of fingers: the overall movement of the limb and hand is correct, but the finger posture is wrong.
4. Spatial orientation error: the arm moves in the wrong direction or in the wrong plane.
5. Spatial error of movement endpoint: the movement endpoint is not reproduced correctly.
6. Static-dynamic spatial error: static posture is produced in response to a dynamic action, or a dynamic movement is produced when the expected target action involves rhythmic or repetitive movement.
7. Kinematic error: movement is in the wrong direction, different velocity, and different trajectory.
8. Prototypicalization: a prototypical version of the action is performed (this applies only to MF actions).
9. Visuosemantic: the action is visually similar and semantically related to the target action (this applies only to MF actions).
10. Perserveration: a movement composed of a combination of gestures previously presented or executed.
11. Global perserveration: an action previously presented or executed is reproduced instead of the target action.
12. Lexicalization: a meaningful action, visual similar to the meaningless target action (but not included in the list), is produced (this applies only to ML actions).
13. Substitution: a visual similar meaningful action (not included in the list) is produced instead of the meaningful action that was presented (this applies only to MF actions).
14. First attempt error: there is one first and faulty attempt to imitate the action, which is corrected, and a *correct answer* is provided at last.
15. Crossing, not crossing: correct gesture but occurring in the wrong side of the body space.
